# Non-invasive Global and Regional Myocardial Work Predicts High-Risk Stable Coronary Artery Disease Patients With Normal Segmental Wall Motion and Left Ventricular Function

**DOI:** 10.3389/fcvm.2021.711547

**Published:** 2021-09-28

**Authors:** Jun Zhang, Yani Liu, Youbin Deng, Ying Zhu, Ruiying Sun, Shirui Lu

**Affiliations:** Department of Medical Ultrasound, Tongji Medical College, Tongji Hospital, Huazhong University of Science and Technology, Wuhan, China

**Keywords:** echocardiography, regional myocardial work, high-risk, stable coronary artery disease, global myocardial work

## Abstract

**Background:** Previous studies suggested that myocardial work (MW) may identify abnormalities in the left ventricular (LV) function and establish a more sensitive index for LV dysfunction at the early stage. This study aimed to explore the value of global and regional MW parameters in predicting high-risk stable coronary artery disease (SCAD) patients with normal wall motion and preserved LV function.

**Patients and Methods:** A total of 131 patients, who were clinically diagnosed as SCAD with normal wall motion and LV function, were finally included in this study. Global MW parameters, including global work index (GWI), global constructive work (GCW), global waste work (GWW), and global work efficiency (GWE) were measured with non-invasive LV pressure-strain loops constructed from speckle-tracking echocardiography. Regional myocardial work index (RWI) and work efficiency (RWE) were also calculated according to the perfusion territory of each major coronary artery. All patients underwent coronary angiography and were divided into the high-risk SCAD group, the non-high-risk SCAD group, and the No SCAD group according to the range and degrees of coronary arteries stenosis.

**Results:** The global longitudinal strain (GLS), GWI and GCW were statistically different (*P* < 0.001) among the three groups. In the high-risk SCAD group, GLS, GWI, and GCW were significantly lower than the other two groups (*P* < 0.05). Receiver operating characteristic analysis demonstrated GWI and GCW could predict high-risk SCAD at a cutoff value of 1,808 mm Hg% (sensitivity, 52.6%; specificity, 87.8%; predictive positive value, 76.3%; predictive negative value, 69.9%) and 2,308 mm Hg% (sensitivity, 80.7%; specificity, 64.9%; predictive positive value, 63.3%; predictive negative value, 80.0%), respectively. Multivariate analyses showed that carotid plaque, decreased GWI, and GCW was independently related to high-risk SCAD. The cutoff values of RWI_LAD_, RWI_LCX_, and RWI_RCA_ were 2,156, 1,929, and 1,983 mm Hg% in predicting high-risk SCAD, respectively (*P* < 0.001). When we combined RWI in two or three perfusion regions, the diagnostic performance of SCAD was improved (*P* < 0.001).

**Conclusions:** Both global and regional MW parameters have great potential in non-invasively predicting high-risk SCAD patients with normal wall motion and preserved LV function, contributing to the early identification of high-risk patients who may benefit from revascularization therapy.

## Introduction

Cardiovascular disease remains the leading cause of death over the world (1), and stable coronary artery disease (SCAD) is a major public health burden (2). Although lifestyle modifications, control of coronary artery disease (CAD) risk factors, and drugs contribute to improving the prognosis in SCAD patients (2, 3), high-risk SCAD patients have a significantly worse prognosis and increased cardiovascular events (4, 5). High-risk SCAD is defined as left main coronary artery diameter stenosis ≥50%, 3-vessel disease (diameter stenosis ≥70%), or 2-vessel disease involving the proximal left anterior descending artery (LAD) (5–7). In these patients, expeditious revascularization has been demonstrated to improve clinical outcomes, exercise capacity, and quality of life more effectively (8, 9). Therefore, it is critical to early and accurate identification of high-risk SCAD patients in clinical practice.

Transthoracic echocardiography is the commonly used non-invasive imaging method for patients with suspected coronary artery stenosis or myocardial ischemia (10). However, routine echocardiography often failed to identify SCAD patients by visually detecting regional wall motion abnormalities (RWMA) (11). Furthermore, detecting RWMA on transthoracic echocardiography is subjective and highly operator and image quality dependent (12). In this situation, speckle-tracking echocardiography was recommended for the early identification of global and regional myocardial dysfunction (13, 14). Although previous studies have confirmed that global longitudinal strain (GLS) is a sensitive parameter in detecting mild systolic dysfunction, it is influenced by LV loading conditions and cannot provide information regarding the efficiency of the ventricle (15).

A novel non-invasive LV pressure-strain loop was firstly developed by Russell et al. which was constructed from speckle-tracking echocardiography (16). With this pressure-strain loop, multiple myocardial work (MW) parameters are obtained for evaluating LV global and regional myocardial function. A previous study demonstrated that reduced MW could identify patients with acute coronary artery occlusion with high sensitivity and specificity, not affected by LV afterload (17). However, the diagnostic value of MW indices, especially the regional MW, in detecting patients with high-risk SCAD and normal wall motion was not fully elucidated. This study aimed to explore the value of global and regional MW parameters in predicting high-risk SCAD patients with normal segmental wall motion and preserved LV function.

## Materials and Methods

### Study Population

Two hundred and five consecutive patients with clinically suspected SCAD were enrolled from August 2018 to December 2019. The inclusion criteria included: (1) chest pain or exertional dyspnea related to myocardial ischemia according to a comprehensive clinical investigation including location, character, duration, and relationship to exertion and other exacerbating or relieving factors; (2) no changes in frequency, duration, precipitating causes or relief for at least 2 months; (3) no evidence of recent myocardial damage. All patients underwent transthoracic echocardiography combined with speckle tracking analysis. Coronary angiography was performed within 3 days after the completion of echocardiography, according to ACC/AHA guidelines (18). The exclusion criteria included: (1) a history of myocardial infarction or revascularization therapy, acute coronary syndrome; (2) LV ejection function (LVEF) <50%; (3) presence of RWMA; (4) other heart diseases including myocardiopathy, valvular heart disease, and congenital heart disease; (5) presence of atrial fibrillation, frequent ventricular or supraventricular ectopy or wide QRS on the electrocardiogram; (6) suboptimal echocardiographic image quality that may influence the analysis of MW or other echocardiographic parameters. A total of 131 patients were finally included in this study. All study procedures were in accordance with the Declaration of Helsinki and the ethical standards of the responsible committee on human experimentation of our hospital. Written informed consent was obtained from all patients.

### Transthoracic Echocardiography

Transthoracic echocardiography was performed by experienced doctors using GE Vivid E95 ultrasound equipment (GE Vingmed Ultrasound, Horten, Norway) with an M5Sc transducer (1.7–3.4 MHz) and a high frame rate (above 70 frame/s). Patients were scanned in the left lateral decubitus position. Parasternal long-axis view, short-axis views (at the basal, middle, and apical levels), and 3 standard apical views (4-chamber, 2-chamber, and apical long-axis) were acquired. Brachial artery systolic was measured simultaneously using a properly sized cuff sphygmomanometer. Three cardiac cycles were stored in the cine-loop format for analysis.

### MW Analysis

All images were digitally stored on the ultrasound system and analyzed offline using EchoPac (GE Healthcare, Horten, Norway, Version 203). Echocardiographic images were interpreted by two experienced doctors blind to each other's findings and clinical information. According to the recommendations of a joint publication of the American Society for Echocardiography and the European Association of Cardiovascular Imaging (19, 20), we performed standard measurements for echocardiographic parameters. Automated functional imaging automatically selected three best matched dynamic images of standard apical views (including long-axis, four-chamber, and two-chamber) in three cardiac cycles, outlined the myocardial border of each wall of the left ventricle to form a region of interest, and then tracked the myocardial movements in the region of interest. In the case of poor tracking, the border of the region of interest can be adjusted manually. LV 17-segment bull's eye diagrams related to longitudinal strain were automatically obtained.

**Figure 1:**
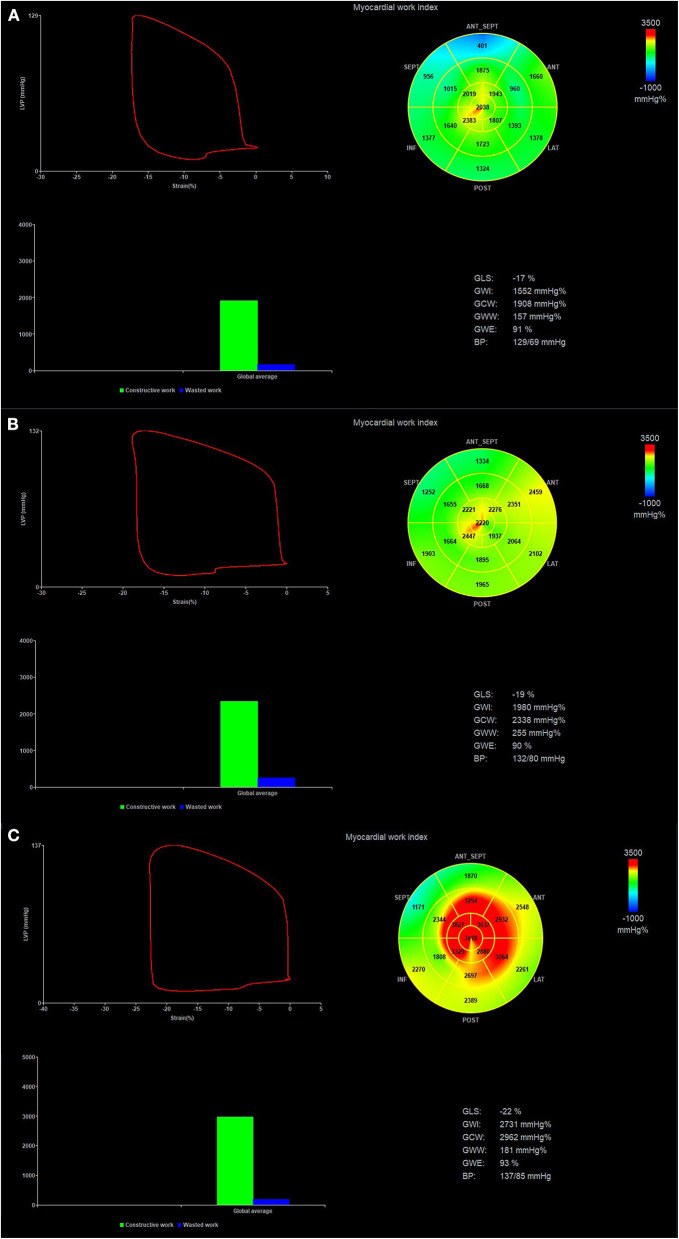
Pressure-strain loop and values of myocardial work in patients with high-risk SCAD **(A)**, non-high-risk SCAD **(B)** and No CAD **(C)**. Regional myocardial work index in 17-segments at Bull's eye diagram was represented. SCAD, stable coronary artery disease.

The LV GLS was the average value from the 17-segment peak systolic longitudinal strain. The brachial cuff systolic pressure was assumed to be equal to the peak systolic LV pressure. Then non-invasive LV pressure curve was constructed according to the duration of isovolumic and ejection phases defined by the timing of aortic and mitral valve opening and closing events on two-dimensional echocardiography (21). The following parameters were calculated: (1) global work index (GWI): total work within the area of the LV pressure-strain loop from mitral valve closing to mitral valve opening; (2) global constructive work (GCW): work performed by the LV contributing to LV ejection during systole, which is the sum of work by the myocytes shortening during systole and the myocytes lengthening during isovolumic relaxation phase; (3) global waste work (GWW): work performed by the LV that does not contribute to LV ejection, which is the sum of work by lengthening of myocytes during systole and shortening during the isovolumic relaxation phase; (4) global work efficiency (GWE): GCW/(GCW+GWW) (Figure 1).

According to AHA's recommendation (22), classification of the perfusion regions of LAD, left circumflex artery (LCX), and right coronary artery (RCA) was divided in the LV 17-segment model. Regional myocardial work index (RWI) and myocardial work efficiency (RWE) (Figure 1) were calculated as the average value in segments belonging to the theoretical perfusion territory of each major coronary artery.

### Coronary Angiography

All patients underwent coronary angiography within 3 days after the completion of echocardiography, according to ACC/AHA guidelines (18). Coronary angiography was performed using the standard technique from the percutaneous femoral approach by two experienced interventionists. All patients were grouped based on the results of angiography. Narrowing of ≥50% in the left main coronary artery and ≥70% in one or several of the major coronary arteries was considered CAD. No CAD was defined as patients without significant coronary stenosis. High-risk SCAD was defined as left main coronary artery diameter stenosis ≥50%, 3-vessel disease (diameter stenosis ≥70%), or 2-vessel disease involving the proximal LAD. Non-high-risk SCAD was defined as patients with ≥70% stenosis in one or two coronary arteries who were excluded from high-risk SCAD.

### Laboratory Examination

Peripheral venous blood samples were collected to determine N-terminal pro-brain natriuretic peptide and cardiac troponin I. According to the inspection project, manual and reagent description in our hospital, the reference range of N-terminal pro-brain natriuretic peptide was: 5.0–97.3 pg/ml (18–44 years old); 5.0–121.0 pg/ml (45–54 years old); 5.0–198.0 pg/ml (55–64 years old); 5.0–285.0 pg/ml (65–74 years old); 5.0–526.0 pg/ml (elder than 75 years). The cardiac troponin level was referred to the 99th percentile upper reference limit in a healthy population, i.e., ≤26.3 pg/ml.

### Electrocardiogram

All patients underwent 12-lead electrocardiogram. An experienced clinician completed the analysis of ECG results. According to the guideline “Recommendations for the Standardization and Interpretation of the Electrocardiogram” (23), ST-T changes were defined as any deviation of the ST-segment below the baseline, while T-wave abnormalities were defined as any negative deflection of the T-wave below the baseline.

### Carotid Artery Ultrasonography

Carotid artery ultrasonography was performed by a GE Vivid E95 ultrasound equipment (GE Vingmed Ultrasound, Horten, Norway) with a 9L transducer (4–8 MHz). A carotid plaque was defined as a focal structure that encroached into the lumen by at least 0.5 mm or 50% of the surrounding intima-media thickness value, or that had a thickness >1.5 mm as measured from the media–adventitia interface to the intima–lumen interface (24).

### Reproducibility Analysis

Measurements of GWI, GCW, GWW, and GWE were repeated in 20 randomly selected data sets to test their reliability. The observer, blind to previous analysis results, measured these parameters twice 2 weeks apart to assess intra-observer variability. Inter-observer variability was evaluated between two independent observers blind to each other's results. Both observers were blind to laboratory examination, electrocardiography, coronary angiography results, and any other patient's medical chart.

### Statistical Analysis

Statistical analysis was performed using SPSS Version 22.0 (IBM Corporation, Armonk, NY, USA) and MedCalc Version 18.11.3 (MedCalc Software, Ostend, Belgium). Continuous variables were expressed as mean ± standard deviation, and the Shapiro-Wilk test was used to verify whether the continuous variables met the normal distribution. Categorical data were summarized as percentages and statistically analyzed with the χ^2^-test or Fisher's exact test. If continuous variables obeyed the normal distribution, the differences among the three groups were analyzed using the one-way analysis of variation (ANOVA) test and the Bonferroni correction for pairwise comparisons between the data in each group. The Kruskal-Wallis rank-sum test was used for variables that did not obey the normal distribution. All pairwise methods were used for further pairwise comparisons between the two groups. Receiver operating characteristic (ROC) analysis was used to investigate the predictive value of each parameter in detecting high-risk SCAD. From ROC analysis, areas under the curve (AUC) and 95% confidence intervals were obtained. By maximization of Youden's index, the optimal cutoff values with specificity or sensitivity were calculated. Comparison of AUC was performed using the method of DeLong in MedCalc. For multivariable logistic regression analyses, variables with significant *P*-values on univariable analyses (*P* < 0.1) were included in the models to detect independent risk factors for predicting high-risk SCAD. Intra-observer and inter-observer reproducibility for MW parameters was assessed using Bland–Altman analysis. All results were considered statistically significantly different at *P* < 0.05.

## Results

### Patient Characteristics

A total of 131 patients were finally evaluated in this study. The detailed clinical characteristics were summarized in Table 1. In comparison with the no CAD group, more male patients were in non-high-risk and high-risk SCAD groups [24 (80.0%) vs. 24 (54.4%), 45 (78.9%) vs. 24 (54.4%), *P* < 0.05]. No significant difference was observed in age, heart rate, blood pressures, and ST-T change across three groups (*P* > 0.05). More patients suffered from carotid plaques in the high-risk SCAD group than the other two groups (*P* < 0.05). Although the levels of cardiac troponin I were not obvious above the normal, significant differences were found in both SCAD groups compared to the no CAD group (*P* < 0.05).

**Table 1:** Baseline clinical characteristics.

**Variable**	**No CAD (*n* = 44)**	**Non-high-risk SCAD (*n* = 30)**	**High-risk SCAD (*n* = 57)**	***P*** **-value**
Age (years)	58 ± 10	60 ± 9	61 ± 10	0.442
Male, *n* (%)	24 (54.5)	24 (80)^*^	45 (78.9)^*^	0.013
Heart rate (beats/min)	69 ± 11	70 ± 10	69 ± 11	0.907
Systolic blood pressure (mmHg)	130 ± 14	131 ± 15	125 ± 16	0.199
Diastolic blood pressure (mmHg)	80 ± 9	76 ± 11	76 ± 11	0.094
Risk factors				
Smoking, *n* (%)	10 (22.7)	9 (30)	26 (45.6)^*^	0.047
Hypertension, *n* (%)	27 (61.4)	21 (70)	39 (68.4)	0.677
Diabetes mellitus, *n* (%)	12 (27.3)	9 (30)	24 (42.1)	0.253
Dyslipidemia, *n* (%)	13 (29.5)	10 (33.3)	19 (33.3)	0.908
Medications				
Aspirin, *n* (%)	34 (77.3)	26 (86.7)	50 (87.7)	0.329
Clopidogrel, *n* (%)	23 (52.3)	25 (83.3)^*^	33 (57.9)^#^	0.019
Low molecular heparin, *n* (%)	1 (2.3)	9 (30)^*^	28 (49.1)^*^	<0.001
Statins, *n* (%)	33 (75)	28 (93.3)^*^	48 (84.2)	0.113
β-blockers, *n* (%)	13 (29.5)	21 (70)^*^	39 (68.4)^*^	<0.001
ACE inhibitors or ARBs, *n* (%)	8 (18.2)	17 (56.7)^*^	30 (52.6)^*^	<0.001
ST-T change, *n* (%)	14 (31.8)	6 (20)	22 (38.6)	0.21
Carotid plaque, *n* (%)	7 (15.9)	7 (23.3)	38 (66.7)^*^^#^	<0.001
NT-proBNP (pg/ml)	60 (26, 103)	73 (30, 224)	129 (66, 335)^*^	0.001
cTnI (pg/ml)	2.4 (1.9, 3.8)	4.3 (2.2, 13.5)^*^	8 (3.4, 105.5)^*^	<0.001

*ARB, angiotensin II receptor antagonist; ACE, angiotensin converting enzyme; NT-proBNP, N-terminal pro-brain natriuretic peptide; cTnI, cardiac troponin I*.

* *P < 0.05 vs. no CAD group*,

# *P < 0.05 vs. non-high-risk SCAD group*.

### Conventional Echocardiographic and Myocardial Work Parameters in Patients With SCAD

The conventional echocardiographic findings are presented in Table 2. The mitral annular septal e′ velocity in the high-risk SCAD group was significantly lower than that in the no CAD group (*P* < 0.05), but no significant difference between non-high-risk and high-risk groups. No significant differences were found regarding LV wall thickness, LV volumes, and LVEF across three groups (*P* > 0.05).

**Table 2:** Conventional echocardiographic parameters in the study population.

**Parameters**	**No CAD (*n* = 44)**	**Non-high-risk SCAD (*n* = 30)**	**High-risk SCAD (*n* = 57)**	***P*** **-value**
IVSd (mm)	9.93 ± 0.95	10.27 ± 0.87	10.05 ± 0.85	0.286
LVPWd (mm)	9.77 ± 0.74	9.97 ± 0.89	9.81 ± 0.79	0.564
LVIDd (mm)	45.30 ± 3.40	46.20 ± 4.44	44.95 ± 4.02	0.368
LVIDs (mm)	29.18 ± 2.65	30.1 ± 3.87	29.32 ± 3.40	0.461
Biplane EDV (mL)	82.16 ± 21.72	83.7 ± 25.07	79.7 ± 20.3	0.697
Biplane ESV (mL)	30.48 ± 9.97	31 ± 10.32	33.04 ± 10.57	0.428
Biplane LVEF (mL)	65.02 ± 5.06	64.13 ± 5.28	64.18 ± 4.94	0.655
Mitral E velocity (cm/s)	74.93 ± 18.65	73.63 ± 17.94	67.18 ± 17.4	0.074
Mitral A velocity (cm/s)	85.05 ± 17.58	83.57 ± 16.23	85.23 ± 17.77	0.907
E/A ratio	0.91 ± 0.27	0.91 ± 0.28	0.82 ± 0.25	0.142
Mitral annular septal e′ velocity (cm/s)	7.77 ± 2.29	6.97 ± 2.06	6.47 ± 1.62^*^	0.005
Mitral annular septal s velocity (cm/s)	8.05 ± 1.61	7.97 ± 1.77	7.49 ± 1.20	0.138
E/E' ratio	10.23 ± 3.28	10.98 ± 2.84	10.68 ± 2.67	0.531

*IVSd, interventricular septal thickness at end-diastole; LVPWd, left ventricular posterior wall thickness at end-diastole; LVIDd, left ventricular internal diameter at end-diastole; LVIDs, left ventricular internal diameter at end-systole; EDV, end-diastolic volume; ESV, end-systolic volume; LVEF, left ventricular ejection fraction*.

* *P < 0.05 vs. no CAD group*.

Among the above three groups, GLS, GWI, and GCW were statistically different (*P* < 0.001), whereas no statistically significant difference in GWW and GWE. GLS, GWI, and GCW in the high-risk SCAD group were significantly lower than those in the other two groups (*P* < 0.05) (Table 3).

**Table 3:** Global and regional myocardial work parameters among the three groups.

**Parameters**	**No CAD (*n* = 44)**	**Non-high-risk SCAD (*n* = 30)**	**High-risk SCAD (*n* = 57)**	***P*** **-value**
Global parameters				
GLS (-%)	20.65 ± 2.43	20.1 ± 2.37	18.35 ± 2.51^*^^#^	<0.001
GWI (mmHg %)	2,142 ± 303	2,070 ± 314	1,752 ± 341^*^^#^	<0.001
GCW (mmHg %)	2,447 ± 352	2,385 ± 309	2,038 ± 370^*^^#^	<0.001
GWW (mmHg %)	146 ± 80	145 ± 90	145 ± 84	0.998
GWE (%)	93 ± 3	93 ± 3	92 ± 3	0.08
Regional parameters				
RWI_LAD_ (mmHg %)	2,159 ± 362	2,128 ± 352	1,808 ± 398^*^^#^	<0.001
RWI_LCX_ (mmHg %)	2,073 ± 360	1,993 ± 338	1,677 ± 407^*^^#^	<0.001
RWI_RCA_ (mmHg %)	2,068 ± 331	1,974 ± 323	1,660 ± 322^*^^#^	<0.001
RWE_LAD_ (%)	92 ± 4	92 ± 4	90 ± 5	0.147
RWE_LCX_ (%)	94 ± 3	94 ± 3	93 ± 6	0.228
RWE_RCA_ (%)	94 ± 4	94 ± 3	93 ± 4	0.253

*GLS, global longitudinal strain; GWI, global work index; GCW, global constructive work; GWW, global waste work; GWE, global work efficiency; RWI_LAD_, RWI_LCX_, RWI_RCA_, myocardial work index in region belonging to the theoretical perfusion territory of left anterior descending artery, left circumflex artery, and right coronary artery, respectively; RWE_LAD_, RWE_LCX_, RWE_RCA_, myocardial work efficiency in region belonging to the theoretical perfusion territory of left anterior descending artery, left circumflex artery, and right coronary artery, respectively*.

* *P < 0.05 vs. no CAD group*,

# *P < 0.05 vs. the non-high-risk SCAD group*.

Regional MW parameters are presented in Table 3. RWI_LAD_, RWI_LCX_, and RWI_RCA_ were significantly lower in patients with high-risk SCAD than that in the no CAD and non-high-risk SCAD group (*P* < 0.001). There was no significant difference in RWE_LAD_, RWE_LCX_, and RWE_RCA_ across the three groups (*P* > 0.05).

### Predictive Value of Global and Regional Myocardial Work Parameters for High-Risk SCAD

**Figure 2:**
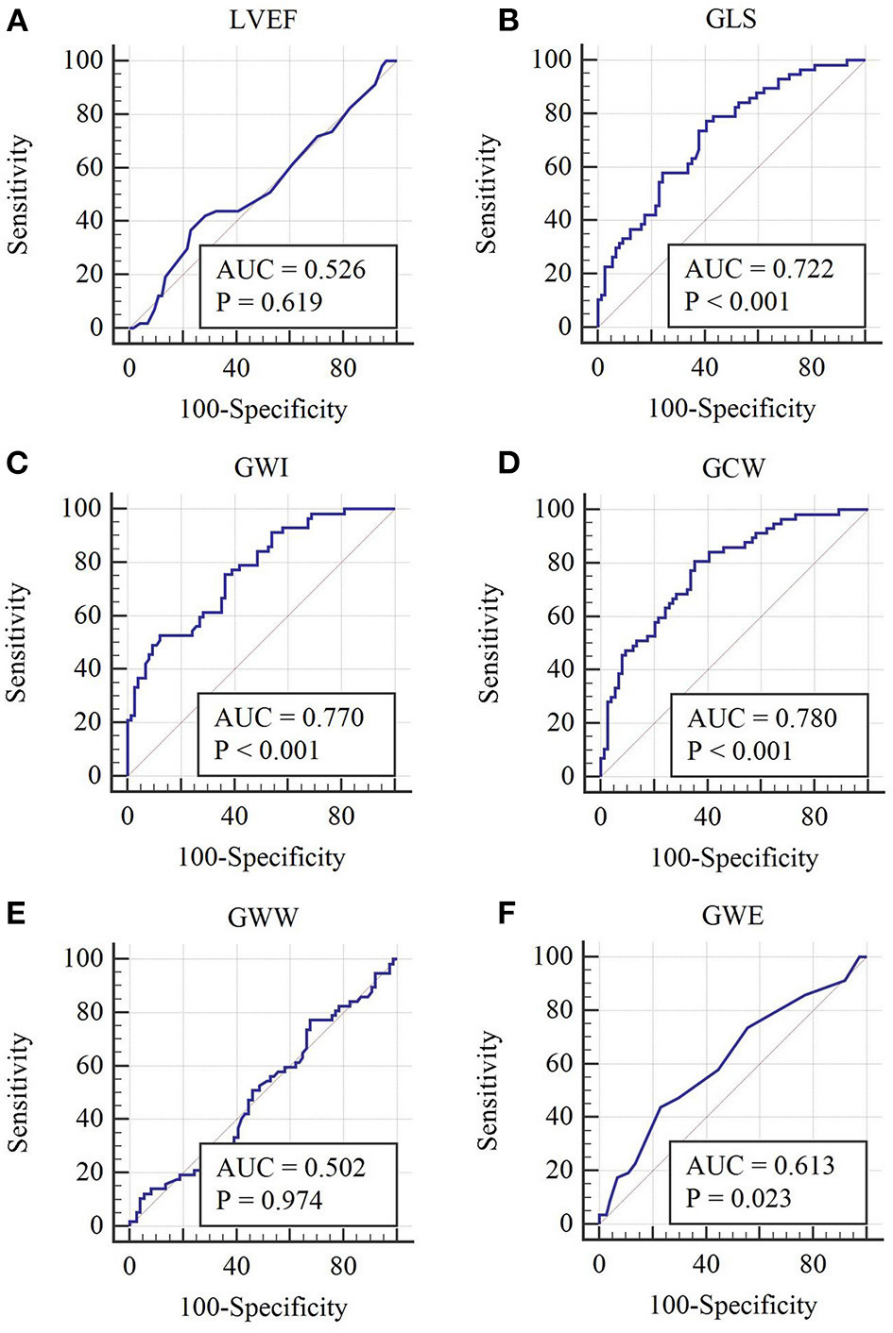
Receiver operating characteristic curves of each global MW parameter. **(A–F)** Receiver operating characteristic curves of LVEF, GLS, GWI, GCW, GWW, and GWE, respectively.

ROC analyses of multiple parameters to predict high-risk SCAD are illustrated in Figure 2 and Table 4. Among the global parameters, GCW were superior to LVEF, GWW, and GWE (AUC = 0.780 [GCW] vs. 0.526 [LVEF], *P* < 0.01; 0.780 [GCW] vs. 0.502 [GWW], *P* < 0.01; 0.780 [GCW] vs. 0.613 [GWE], *P* < 0.01) in detecting high-risk SCAD (Figure 2 and Table 4). Also, GWI was superior LVEF, GWW, and GWE (AUC = 0.770 [GWI] vs. 0.526 [LVEF], *P* < 0.01; 0.770 [GWI] vs. 0.502 [GWW], *P* < 0.01; 0.770 [GWI] vs. 0.613 [GWE], *P* < 0.01) in detecting high-risk SCAD (Figure 2 and Table 4). The cutoff values were 1,808 mm Hg% for GWI with a sensitivity 52.6% and specificity 87.8% (the predictive positive and negative values were 76.3% and 69.9%, respectively), and 2,308 mm Hg% for GCW with sensitivity 80.7% and specificity 64.9% (the predictive positive and negative values were 63.3% and 80.0%, respectively). The AUC of GWI and GCW was slightly higher than GLS, but these relations did not reach statistical significance in this cohort (AUC = 0.770 [GWI] vs. 0.722 [GLS], *P* > 0.05; 0.780 [GCW] vs. 0.722 [GLS], *P* > 0.05; 0.770 [GWI] vs. 0.780 [GCW], *P* > 0.05).

**Table 4:** Receiver operating characteristic curve analysis of LVEF and global myocardial work parameters to identify high-risk SCAD.

**Parameters**	**AUC**	**Standard error**	**AUC 95% CI**	***p*** **-value**	**Cutoff value**	**Sensitivity (%)**	**Specificity (%)**
LVEF (%)	0.526	0.0517	0.437–0.614	0.619	61	36.8	77.0
GLS (-%)	0.722	0.0442	0.637–0.797	<0.001	19.97	77.2	59.5
GWI (mmHg %)	0.770	0.0405	0.689–0.839	<0.001	1,808	52.6	87.8
GCW (mmHg %)	0.780	0.0401	0.700–0.848	<0.001	2,308	80.7	64.9
GWW (mmHg %)	0.502	0.0514	0.413–0.590	0.974	157	77.2	32.4
GWE (%)	0.613	0.0498	0.524–0.697	0.023	91	43.9	77.0

*AUC, area under the curve; LVEF, left ventricular ejection fraction; GLS, global longitudinal strain; GWI, global work index; GCW, global constructive work; GWW, global waste work; GWE, global work efficiency*.

**Figure 3:**
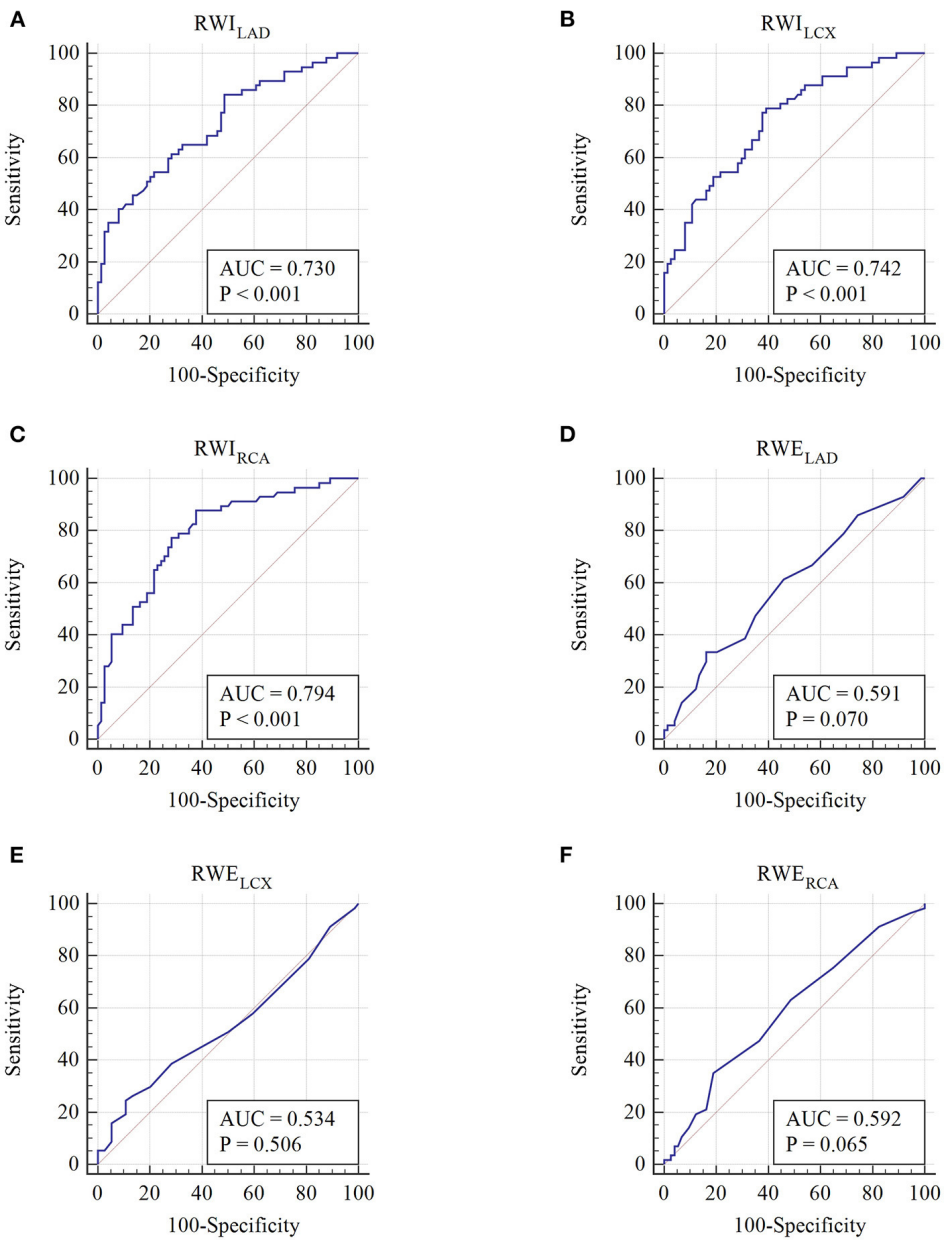
Receiver operating characteristic curves of each regional MW parameter. **(A–F)** Receiver operating characteristic curves of RWILAD, RWILCX, RWIRCA, RWELAD, RWELCX, and RWERCA, respectively.

As shown in Figure 3 and Table 5, the AUC of RWI_LAD_, RWI_LCX_, and RWI_RCA_ in predicting high-risk SCAD were 0.730, 0.742, and 0.794, respectively (*P* < 0.001 for all). No statistical significance of RWE_LAD_, RWE_LCX_ and RWE_RCA_ in predicting high-risk SCAD was found (*P* > 0.05). When combining regional MW in two or three regions, the diagnostic performance in predicting high-risk SCAD was improved (*P* < 0.001) (Table 5).

**Table 5:** Receiver operating characteristic curve analysis of regional myocardial work parameters to identify high-risk SCAD.

**Parameters**	**AUC**	**Standard error**	**AUC 95% CI**	***p*** **-value**	**Cutoff value**	**Sensitivity (%)**	**Specificity (%)**
RWI_LAD_ (mmHg %)	0.730	0.0443	0.646–0.804	<0.001	2,156	84.2	51.3
RWI_LCX_ (mmHg %)	0.742	0.0429	0.659–0.815	<0.001	1,929	79.0	60.8
RWI_RCA_ (mmHg %)	0.794	0.0395	0.714–0.859	<0.001	1,983	87.7	62.2
RWI_LAD_+RWI_LCX_	0.754	0.0419	0.672–0.825	<0.001	–	50.9	87.8
RWI_LAD_+RWI_RCA_	0.793	0.0394	0.714–0.859	<0.001	–	78.9	70.3
RWI_LCX_+WI_RCA_	0.802	0.0385	0.723–0.866	<0.001	–	66.7	83.8
RWI_LAD_+RWI_LCX_+RWI_RCA_	0.805	0.0381	0.726–0.869	<0.001	–	78.9	70.3

*RWI_LAD_, myocardial work index in region belonging to the theoretical perfusion territory of left anterior descending artery; RWI_LCX_, myocardial work index in region belonging to the theoretical perfusion territory of left circumflex artery; RWI_RCA_, myocardial work index in region belonging to the theoretical perfusion territory of right coronary artery*.

### Univariate and Multivariate Analyses of Risk Factors Related to High-Risk SCAD

The univariate logistic analysis demonstrated no significant correlation between age, comorbidities, ST-T change, GWW, and GWE, and the high-risk SCAD. The further multivariate logistic analysis (model 1) demonstrated that carotid plaque and decreased GWI were independently related to high-risk SCAD (carotid plaque: odds ratio 7.717, 95% confidence interval 3.124–19.065, *P* < 0.001; decreased GWI: odds ratio 7.305, 95% confidence interval 2.529–21.098, *P* < 0.001, Table 6). When reduced GCW was added in the model instead of GWI (model 2), carotid plaque and decreased GCW were independently related to high-risk SCAD (carotid plaque: odds ratio 7.510, 95% confidence interval 3.015–18.704, *P* < 0.001; decreased GCW: odds ratio 5.828, 95% confidence interval 2.242–15.152, *P* < 0.001, Table 6).

**Table 6:** Univariate and multivariate analyses of risk factors for high-risk SCAD.

**Variable**	**Univariate analysis**			**Multivariate analysis (Model 1)**			**Multivariate analysis (Model 2)**		
	**OR**	**95% CI**	***P*** **-value**	**OR**	**95% CI**	***P*** **-value**	**OR**	**95% CI**	***P*** **-value**
Male	2.031	0.917–4.502	0.081	2.356	0.721–7.696	0.156	1.720	0.549–5.387	0.352
Age	0.979	0.944–1.015	0.245						
Smoking	2.428	1.161–5.075	0.018	1.352	0.492–3.716	0.558	1.702	0.612–4.733	0.308
Hypertension	1.174	0.563–2.447	0.669						
Diabetes mellitus	1.835	0.885–3.806	0.103						
Dyslipidemia	1.109	0.530–2.321	0.784						
ST-T change	1.697	0.810–3.557	0.161						
Carotid plaque	8.571	3.847–19.096	<0.001	7.717	3.124–19.065	<0.001	7.510	3.015–18.704	<0.001
Increased NT-proBNP	2.346	1.040–5.292	0.04	0.677	0.198–2.315	0.534	0.809	0.253–2.593	0.722
Increased cTnI	5.385	1.836–15.795	0.002	3.050	0.727–12.804	0.128	2.298	0.534–9.893	0.264
Decreased GWI	7.480	3.136–17.841	<0.001	7.305	2.529–21.098	<0.001			
Decreased GCW	6.923	3.124–15.343	<0.001				5.828	2.242–15.152	<0.001
Increased GWW	1.625	0.739–3.570	0.227						
Decreased GWE	1.891	0.762–4.695	0.170						

*NT-proBNP, N-terminal pro-brain natriuretic peptide; cTnI, cardiac troponin I; GWI, global work index; GCW, global constructive work; GWW, global waste work; GWE, global work efficiency*.

### Inter-observer and Intra-Observer Variability Analyses

**Figure 4:**
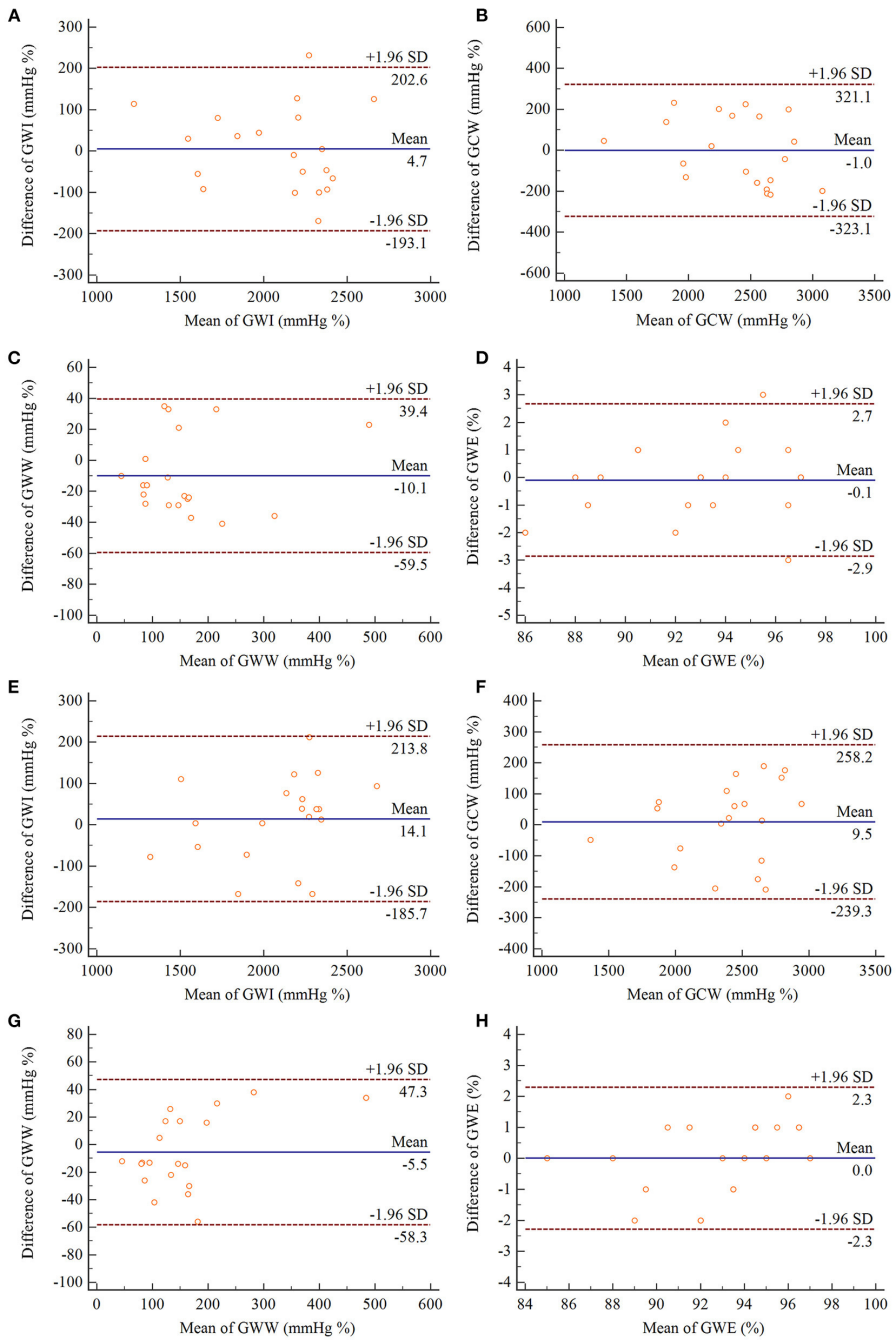
Bland–Altman analyses of intra-observer **(A–D)** and inter-observer **(E–H)** reproducibility for measurements of GWI, GCW, GWW, GWE. In each panel, the solid line represents mean difference, and the broken lines represent the 95% limits of agreement. GWI, global work index; GCW, global constructive work; GWW, global waste work; GWE, global work efficiency.

All MW parameters exhibited excellent intra- and inter-observer reproducibility, and results of the Bland–Altman analysis were illustrated in Figure 4. The ICCs for inter-observer and intra-observer variability for MW parameters are listed in Table 7.

**Table 7:** Inter- and intra-observer variability for myocardial work parameters.

	**Interobserver variability**			**Intraobserver variability**		
**Parameters**	**ICC**	**95% CI**	***p*** **-value**	**ICC**	**95% CI**	***p*** **-value**
GWI (mmHg %)	0.959	0.900–0.983	<0.001	0.965	0.913–0.986	<0.001
GCW (mmHg %)	0.951	0.880–0.980	<0.001	0.933	0.837–0.973	<0.001
GWW (mmHg %)	0.960	0.904–0.984	<0.001	0.965	0.910–0.986	<0.001
GWE (%)	0.938	0.850–0.975	<0.001	0.915	0.798–0.965	<0.001

*GWI, global work index; GCW, global constructive work; GWW, global waste work; GWE, global work efficiency; ICC, intraclass correlation coefficient*.

## Discussion

Our findings are illustrated as follows, (1) Both GWI and GCW could identify high-risk SCAD accurately with good sensitivity and specificity. (2) In addition, the regional myocardial work index in each perfusion territory of the three primary coronary arteries showed excellent diagnostic performance in predicting high-risk-SCAD. (3) Multivariate analyses found that carotid plaque, decreased GWI, and GCW was independently related to high-risk stable coronary artery disease. This study confirmed that the non-invasive global and regional MW parameters are of great value in facilitating accurate identifying high-risk SCAD, especially in those without visually detectable wall motion abnormalities and LV dysfunction.

SCAD is a major public health burden worldwide, and its prevalence is as high as 14% in middle and advanced ages. Besides, the annual incidence of SCAD ranges from around 1% in middle-aged individuals up to 4% in the elderly (3). In clinic, the apparently stable patients are heterogeneous. Some high-risk patients may have a greater probability of having major adverse cardiovascular events (5). Studies showed that high-risk SCAD benefits more from coronary revascularization compared with medical therapy (8, 9). It is pretty essential to identify those patients at higher risk and further optimize their therapeutic management. Transthoracic echocardiography is considered the first-line imaging modality for diagnosing coronary heart disease by visually detecting segmental wall motion abnormalities. However, the limited sensitivity and specificity have previously been criticized due to normal segmental wall motion and LV function at rest in most patients with SCAD (11). This phenomenon may be attributed to well-developed coronary collateral circulation and coronary flow reserve (10, 25). Furthermore, detecting RWMA on transthoracic echocardiography is subjective and highly operator and image quality dependent. Although coronary angiography can determine the severity of coronary artery stenosis, it is not an ideal strategy for screening out high-risk patients because of its invasive procedure and high expenses.

In the previous studies, the longitudinal strain is proved to be superior to LVEF in early detecting myocardial dysfunction caused by ischemia (13, 26, 27). However, recent studies showed that LV myocardial strain was load-dependent. It could decrease significantly when the LV afterload was obviously elevated (27, 28). Nowadays, non-invasive approaches independent of afterload were developed to identify myocardial dysfunction by investigating myocardial energetics and metabolism (29–32). Unlike the myocardial power and power efficiency derived from cardiac magnetic resonance, which was partly based on wall stress analysis according to the Laplace law, non-invasive myocardial work indices measured with echocardiography come from the LV pressure-volume loop in principle. For a long time, LV pressure-volume loop was considered the gold standard to measure myocardial energetics, providing a sensitive indicator in identifying cardiac dysfunction. However, this invasive procedure obtained by left heart catheterization is not feasible in practical application (33–35). According to the same principle, a more convenient and efficient method, myocardial work (MW), is recently introduced for assessing global and regional myocardial function by the pressure-strain loop (16, 17, 36). Since Russell et al. first introduced this methodology in detail and validated it by animal and clinical studies (16), MW was widely used in various cardiovascular diseases (37).

A previous study showed that MW could identify patients with acute coronary artery occlusion with high sensitivity and specificity (17). We further explore the predictive value of multiple MW parameters in SCAD patients with normal segment wall motion and LV function in the present study. We found both GWI and GCW were superior to LVEF, GWW, and GWE in detecting the high-risk patients with SCAD at rest. In Edwards's study (38), the global MW was the most powerful predictor for significant CAD (AUC = 0.786) and was superior to GLS (AUC = 0.693). The optimal cutoff global MW value in predicting significant CAD was 1,810 mm Hg% (sensitivity, 92%; specificity, 51%). Although the AUCs of GWI and GCW were slightly higher than GLS, these relations did not reach statistical significance in the present cohort. On the one hand, a larger sample size is needed. And on the other hand, it indicated that GLS was still an important indicator in diagnosing high-risk SCAD, while MW was supplemented to collaborative assessment. Chan et al. considered that MW parameters were not likely to replace GLS but may add incremental value to existing strain evaluation (39). Moreover, in our study, the optimal cutoff GWI, GCW and GLS value to predict high-risk SCAD was 1,808 mm Hg% (sensitivity, 52.6%; specificity, 87.8%), 2,308 mm Hg% (sensitivity, 80.7%; specificity, 64.9%), and −19.97% (sensitivity, 77.2%; specificity, 59.5%). As the total work within the area of the LV pressure-strain loop, GWI only reflected a value during the whole cardiac cycle. However, there were complex dynamic changes between myocardial contraction with intraventricular pressure and LV geometry during the cardiac cycle (39). This may be the reason why the sensitivity of GWI is inferior to GCW and GLS to predict high-risk SCAD. Because GCW took into account only positive work, it reflected the contractile function of the myocardium better. Recent studies showed that GCW was the most sensitive MW parameter in patients with HCM and acute anterior myocardial infarction treated by percutaneous coronary intervention in assessing global and regional myocardial function (40, 41). It should be noted that the sensitivity and specificity of GCW were slightly higher than GLS, indicating that GCW may have the better ability to identify high-risk SCAD. A larger sample size is needed in further study.

Pieter et al. reported that RWE and RWI values were decreased in the anterior wall (supplied by the LAD with complete occlusion), with compensatory increases in the lateral wall in a patient with ST-segment elevation myocardial infarction (42). These findings showed the potential of regional MW parameters in diagnosing cardiovascular disease. In our study, RWI in each region belonging to the theoretical perfusion territory of three primary coronary arteries showed significant diagnostic efficacy to predict high-risk SCAD. For some patients in our study cohort, RWI decreased, but GWI and GCW remained normal due to the compensation of the myocardium belonging to the non-stenosis artery. This may be attributed to well-developed coronary collateral circulation and coronary flow reserve. When MW values in two or three regions were combined, the diagnostic performance in predicting high-risk SCAD was improved. These findings suggested that regional MW parameters could be used as supplements to global parameters in diagnosing high-risk SCAD.

Identifying patients with high-risk CAD is always a common problem in clinical management. Over the years, the presentation and treatment of CAD have dramatically changed due to various prediction models were worked out. Jang et al. developed a new model based on clinical features, risk factors, and test results in symptomatic outpatients with high-risk CAD before any non-invasive testing (5). In our study, we further explored the predictive value of the non-invasive echocardiographic characteristics. Our results showed that carotid plaque, decreased GWI and GCW were independent risk factors related to high-risk SCAD. Numerous studies have confirmed that carotid plaque was highly correlated with CAD. Carotid intima-media thickness and plaques, neovascularization, calcium-like tissue composition, and other features indicate high-risk CAD and cardiovascular events (43–45). This identification of extracardiac atherosclerosis might be equally valuable for echocardiography. Therefore, these non-invasive imaging parameters could be considered in future prediction models.

As indicated in the present study, non-invasive global and regional MW parameters can be obtained easily with speckle tracking automated functional imaging analysis. Therefore, it hold the promise to be used in clinical practice and to provide the incremental value during the work-up in identifying high-risk SCAD patients. For the suspected SCAD patients, particularly those without visually RWMA and abnormal LVEF, MW contribute early diagnosis by detecting mild systolic dysfunction. Based on the diagnostic assessments, further risk stratification was served to identify high risk ones who will benefit from revascularization. According to our study, both GWI and GCW could predict high-risk SCAD patients with cutoff values of 1,808 and 2,308 mm Hg%, respectively. Besides, regional MW and carotid plaque also should be considered for the identification of high-risk ones. On the contrary, a complete normal non-invasive test result is often associated with a low event risk. In principle, MW parameters overcome the afterload dependency of other echocardiographic parameters. In complex clinical situations, this is particularly useful in identifying cardiac dysfunction due to increased afterload. Further follow-up evaluation of the event risk should be performed to specifically guide clinical treatment and prognosis.

### Limitations

There are still some limitations in the present study. Firstly, although the AUCs of GWI and GCW were higher than that of GLS according to the ROC analysis, we cannot prove the superiority of myocardial work to GLS in identifying high-risk SCAD patients with statistical significance in the present cohort. The underlying reason could be the limited sample size for the strict inclusion and exclusion criteria. Many patients were excluded because of suboptimal image quality and arrhythmia, which influenced the analysis of MW or other echocardiographic parameters. Secondly, MW parameters are derived from a non-invasive pressure-strain loop method. In this method, the pressure was estimated by brachial artery systolic, measured using a properly sized cuff sphygmomanometer. This estimated value may lead to bias in the results. Furthermore, the present study was focused on exploring the diagnostic value of global and regional MW parameters in SCAD patients. We did not compare their value with blood tests, electrocardiograms, and other imaging techniques. Lastly, previous studies confirmed the diagnostic value of stress echocardiography in CAD (46, 47), which contributing to the further risk stratification based on the assessments. Further studies should be performed to explore whether MW parameters could improve the predictive power during stress echocardiography.

## Conclusion

In summary, both GWI and GCW could be used to accurately identify high-risk SCAD patients who may benefit from revascularization therapy. In addition, regional MW parameters could also provide incremental diagnostic information in identifying high-risk SCAD.

## Data Availability Statement

The raw data supporting the conclusions of this article will be made available by the authors, without undue reservation.

## Ethics Statement

The studies involving human participants were reviewed and approved by Tongji Hospital Ethics Committee. The patients/participants provided their written informed consent to participate in this study.

## Author Contributions

JZ drafted the manuscript, which was critically revised and edited by YL. Other authors helped to collect clinical information, recording, and analyzing images. All authors agree to be accountable for all aspects of the work.

## Conflict of Interest

The authors declare that the research was conducted in the absence of any commercial or financial relationships that could be construed as a potential conflict of interest.

## Publisher's Note

All claims expressed in this article are solely those of the authors and do not necessarily represent those of their affiliated organizations, or those of the publisher, the editors and the reviewers. Any product that may be evaluated in this article, or claim that may be made by its manufacturer, is not guaranteed or endorsed by the publisher.

## Supplementary Material

Supplementary Figure 1 Step wise calculation of various MW parameters. **(A)** Global longitudinal strain and segmental longitudinal strain displayed as bull's eye diagram were obtained using automated functional imaging. **(B)** Timing of aortic and mitral valve opening and closing events was confirmed on two-dimensional echocardiography, and brachial cuff systolic pressure was input. **(C)** The pressure-strain loop, various MW parameters, and bull's eye diagram of segmental MW was output. MW, myocardial work.

## Abbreviations

MW: myocardial work
LV: left ventricular
CAD: coronary artery disease
SCAD: stable coronary artery disease
LAD: left anterior descending artery
LVEF: left ventricular ejection function
GLS: global longitudinal strain
GWI: global work index
GCW: global constructive work
GWW: global waste work
GWE: global work efficiency
LCX: left circumflex artery
RCA: right coronary artery
RWI: regional myocardial work index
RWE: regional myocardial work efficiency
ROC: receiver operating characteristic
AUC: area under the curve
RWMA: regional wall motion abnormalities.
