# Supplementary figures and images for: Non-invasive Global and Regional Myocardial Work Predicts High-Risk Stable Coronary Artery Disease Patients With Normal Segmental Wall Motion and Left Ventricular Function
Source: Front Cardiovasc Med. 2021 Sep 28;8:711547. doi: 10.3389/fcvm.2021.711547 (PMC8505723; doi:10.3389/fcvm.2021.711547)

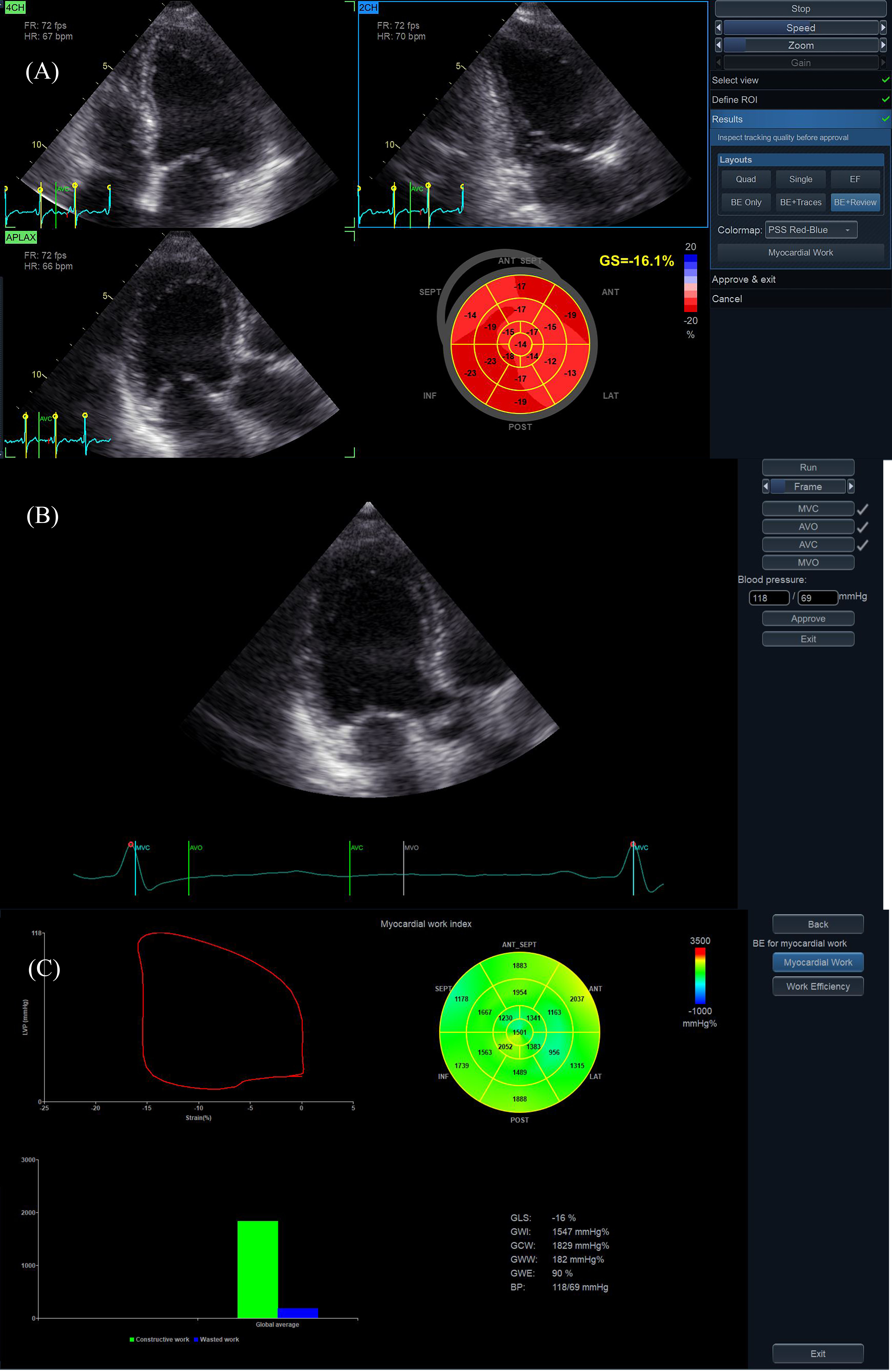

Supplement: Supplementary Figure 1 — Step wise calculation of various MW parameters. (A) Global longitudinal strain and segmental longitudinal strain displayed as bull's eye diagram were obtained using automated functional imaging. (B) Timing of aortic and mitral valve opening and closing events was confirmed on two-dimensional echocardiography, and brachial cuff systolic pressure was input. (C) The pressure-strain loop, various MW parameters, and bull's eye diagram of segmental MW was output. MW, myocardial work. [file Image_1.JPEG]
